# Effects of *Gladiolus dalenii* on the Stress-Induced Behavioral, Neurochemical, and Reproductive Changes in Rats

**DOI:** 10.3389/fphar.2017.00685

**Published:** 2017-09-27

**Authors:** David Fotsing, Gwladys T. Ngoupaye, Agnes C. Ouafo, Stephanie K. J. Njapdounke, Yongabi A. Kenneth, Elisabeth Ngo Bum

**Affiliations:** ^1^Department of Biological Sciences, Faculty of Science, University of Bamenda, Bambili, Cameroon; ^2^Department of Biological Sciences, Faculty of Sciences, University of Ngaoundéré, Ngaoundéré, Cameroon; ^3^Department of Animal Biology and Physiology, Faculty of Science, University of Dschang, Dschang, Cameroon; ^4^Directorate of Research, Catholic University of Cameroon, Bamenda, Cameroon; ^5^Institute of Mines and Petroleum Industries, University of Maroua, Maroua, Cameroon

**Keywords:** biochemical parameters, estrous cycle, *Gladiolus dalenii*, neurochemical, restraint stress

## Abstract

*Gladiolus dalenii* is a plant commonly used in many regions of Cameroon as a cure for various diseases like headaches, epilepsy, schizophrenia, and mood disorders. Recent studies have revealed that the aqueous extract of *G. dalenii* (AEGD) exhibited antidepressant-like properties in rats. Therefore, we hypothesized that the AEGD could protect from the stress-induced behavioral, neurochemical, and reproductive changes in rats. The objective of the present study was to elucidate the effect of the AEGD on behavioral, neurochemical, and reproductive characteristics, using female rats subjected to chronic immobilization stress. The chronic immobilization stress (3 h per day for 28 days) was applied to induce female reproductive and behavioral impairments in rats. The immobilization stress was provoked in rats by putting them separately inside cylindrical restrainers with ventilated doors at ambient temperature. The plant extract was given to rats orally everyday during 28 days, 5 min before induction of stress. On a daily basis, a vaginal smear was made to assess the duration of the different phases of the estrous cycle and at the end of the 28 days of chronic immobilization stress, the rat’s behavior was assessed in the elevated plus maze. They were sacrificed by cervical disruption. The organs were weighed, the ovary histology done, and the biochemical parameters assessed. The findings of this research revealed that *G. dalenii* increased the entries and the time of open arm exploration in the elevated plus maze. Evaluation of the biochemical parameters levels indicated that there was a significant reduction in the corticosterone, progesterone, and prolactin levels in the *G. dalenii* aqueous extract treated rats compared to stressed rats whereas the levels of serotonin, triglycerides, adrenaline, cholesterol, glucose estradiol, follicle stimulating hormone and luteinizing hormone were significantly increased in the stressed rats treated with, *G. dalenii*, diazepam and in co-administration of the plant extract and diazepam treated rats. Moreover stressed rats showed significant changes in estrous cycle phases compared to vehicle control and these changes of the estrous cycle were less in the rats treated with *G. dalenii* compared to the negative control rats. *G. dalenii* extract showed antagonizing effects on the stress-induced reproductive, behavioral, and neurochemical changes. These effects could be related to the bioactive molecules and secondary metabolites like alkaloids and flavonoids in the plant.

## Introduction

Individuals frequently face stressful conditions. Chronic stress consistently activates the hypothalamic–pituitary–adrenal (HPA) axis. Each individual component of the HPA axis exerts deleterious effect on the hypothalamic–pituitary–gonadal axis and subsequently leads to human reproductive failure (Nepomnaschy et al., 2004; Chatterjee et al., 2006). During stress induction, many behavioral, biochemical, and reproductive parameters are altered. The stress-induced alterations have been attributed to an imbalance in the neuroendocrine system (Kenjale et al., 2007). Therefore, assessment of some of the biochemical, endocrinal, and behavioral parameters will serve as an important basis for the evaluation of anti-stress activity (Rai et al., 2003). Biological responses to stress are known to suppress reproductive function across the human life course. For example, hypothalamic amenorrhea, a clinical condition without endocrine or systemic cause, is triggered by metabolic, physical, or psychological stress as well as high stress perception is a risk factor for severe premenstrual pain or ovarian dysfunction (Woods et al., 1998; Kaplan and Manuck, 2004; Genazzani et al., 2006). Impairment of reproductive outcomes is triggered by stress-inducing factors and is more established in women susceptible to a physiological stress response (Brotman et al., 2007). Unlike in the males, the level of corticotropin-releasing factor (CRF) in the female hypothalamus is very important (Fredericksen et al., 1991), therefore, the females HPA axis responds to stress more intensely than the males (Lund et al., 2004). In women in their working environment, persistent stimulation of the HPA axis has been shown to hamper the hypothalamic–pituitary–ovarian axis (Imaki et al., 1996; Fenster et al., 1999). Stress powerfully stimulates the hypothalamus and extra-hypothalamic sites for the release of CRF (Horrocks et al., 1990). Rats with normal estrous cycle restrained inside a cylindrical restrainers (stress induction) exhibit behavioral, neurochemical, and reproductive impairments (Bowman et al., 2001; Sivaprasad et al., 2015).

In the stress-related disorders, the treatments available include the anxiolytics like the benzodiazepines. These treatments have a broad numbers of side effects such as the muscle relaxation, the memory loss, and addiction (Lader and Morton, 1991; Czobor et al., 2010). These limits have developed more interest in the use of natural products to treat stress-related disorders. Several plants have proven anxiolytic-like effects in animal models. For example, *Afrormosia laxiflora* (Benth) Harms (Fabaceae), *Chenopodium ambrosioides* Linn (Chenopodiaceae), *Microglossa pyrifolia* Kuntze (Lam) (Asteraceae), *Mimosa pudica* Linn (Mimosaceae), *Nelsonia canescens* (Acanthaceae), and *Gladiolus dalenii* Van Geel (Iridaceae) (Ngo Bum et al., 2011; Ngoupaye et al., 2013a; Fotsing et al., 2016). *G. dalenii* is plant of the Iridaceae family generally used in Cameroon pharmacopoeia, especially in the West region to cure various ailments like schizophrenia, depression, and headaches. The research conducted by Adejuwon et al. (2013) revealed that uncontrolled consumption of the aqueous extract of *G. dalenii* (AEGD) had toxic effects in male rat’s reproductive parameters notably the spermatozoa. Past studies indicated that *G. dalenii* corm crude extract had antifungal activity (Odhiambo et al., 2010), anticonvulsivant and sedative effects (Ngoupaye et al., 2013b), and antidepressant-like effects in epileptic mice (Ngoupaye et al., 2013a). The data indicated that the ability of *G. dalenii* to antagonize the PTZ-induced seizures could be attributed to its modulatory effects on the GABA_A_ receptor neurotransmission. Also, the antidepressant activity could be mediated through the restoration of the HPA axis activities (Ngoupaye et al., 2013a). Therefore, this study focuses on the effects of the AEGD on the stress-induced behavioral and physiological changes in non-epileptic chronic stressed adult female rats. These effects were assessed using the elevated plus maze test, and measuring the stress markers and the reproductive parameters. The effects of *G. dalenii* were compared to those of diazepam, a benzodiazepine that binds to a specific subunit on the GABA_A_ receptor, inducing anxiolytic effects (Campo-Soria et al., 2006).

## Materials and Methods

### Plant and Aqueous Extract Preparation

The corms of *G. dalenii* were harvested in Babajou in the West region of Cameroon during the month of November 2013 and were identified at the national Herbarium of Yaoundé (number 25742/SRF/Cam). They were cleaned in water to remove dust and mud, dried under ambient air, and ground to get a powder that was used to prepare the AEGD (Fotsing et al., 2016). The aqueous extract was provided by macerating 250 g of the air dried powder of *G. dalenii* in 6 l of distilled water for 72 h at room temperature. The preparation was filtrated and the filtrate was evaporated to dryness in an oven at 35°C and 24.6 g of a brown solid extract was obtained. The yield of the extraction was 9.84%.

### The Animals and Experimental Design

Adult female Wistar albino rats with normal estrous cycle were used in the study. They were provided from the animal unit of the University of Bamenda. These rats were raised in standard conditions (room temperature, 12/12 h light–dark cycle). They were supplied with pellets and water *ad libitum*. The study was carried out in accordance with the Cameroon National Ethical Committee (Ref No. FW-IRB00001954, 22 October 1987 with an authorization number CEI-UDo/909/01/2017/T), and in conformation with the international regulation, minimizing the number of rats used and their suffering. The animals were organized into six groups containing five rats each. The vehicle control group was given distilled water and was kept unstressed, the negative control group and the positive group were respectively treated with the distilled water and the reference substance, diazepam (3 mg/kg) and were stressed. Two experimental groups received two doses of the extract (7.5 or 15 mg/kg), and were stressed. An experimental group was treated in co-administration with the plant extract (15 mg/kg) and diazepam (3 mg/kg) and was stressed. The doses used in the experiment were based on previous study (Ngoupaye et al., 2013a). The experimental rats were adapted in the new environment for 2 weeks and then were stressed. The restraint stress involved confining rats inside individual plastic cylindrical restrainers (21 cm in length × 6 cm in diameter) with ventilated sliding doors at ambient temperature (Bowman et al., 2001; Ngoupaye et al., 2013b; Sivaprasad et al., 2015). This restraint stress procedure was performed 3 h daily for 28 days. Right after the stress period, a vaginal smear was prepared to find out the consequent stage of the estrous cycle. Vaginal smears were obtained by placing a small drop of saline in the vagina with a blunted Pasteur pipette and removing a sample of vaginal cells which were immediately observed microscopically under low magnification (Baron and Brush, 1979; Saraswathi et al., 2010; Sivaprasad et al., 2015). The rats of the vehicle control group were taken to a different experimental room and kept in plastic-box cages unstressed. After the restraint stress sequences, the behavior was evaluated in the elevated plus maze before the rats were sacrificed by cervical disruption. The blood was collected in EDTA tubes and the biochemical parameters measured, while the organs were freed from connective tissues and weighed. The ovaries imbedded in paraffin were cut into 4 μm sections and stained with hematoxylin–eosin for histological analysis.

### Behavioral Assessment

The behavioral test was done with the elevated plus maze (Pellow et al., 1985). The elevated plus maze was placed in an isolated room, far from any irrelevant interference of scents, movement, or noises. The arms of the maze were approximately 90 cm above the floor which was covered by foam rubber. At the beginning of each session, the rat was placed in the central area facing the open arms of the maze and was allowed to explore the maze freely during 5 min. The entries and the time spent in the different arms were recorded. The data were used to calculate the percentage of entries and time spent for each arm. After the assessment of a rat’s behavior, the maze was cleaned with alcohol.

### Biochemical Parameters

Blood was centrifuged at 3,000 rpm for 20 min at 4°C, and 1 ml aliquot of plasma was transferred to 1.5 ml Eppendorf vials and kept at -20°C. Plasma serotonin, adrenaline, triglycerides, glucose, cholesterol, estradiol, prolactin, follicle stimulating hormone (FSH), luteinizing hormone (LH), progesterone, and corticosterone were measured using commercially available immunoassay kits (Human Gesellschaft für Biochemica und Diagnostica mbH, Wiesbaden, Germany).

### Statistical Analysis

Statistical analysis was done using the software program Statgraphics 11.0. All data are presented as mean ± SEM. Analysis of variance (ANOVA) was done for different groups and means were separated by Newman–Keuls *post hoc* test and corrected with Bonferroni multiple comparisons test at 5% confident limit.

## Results

### Effect of AEGD on Stress-Induced Behavioral Change

Our results indicated that in the elevated plus maze, the chronic restraint stress (CRS) caused a decrease (though not significant) of the number open arms entries and a significant (*p* ≤ 0.05) decrease of the open arm time. The AEGD (7.5 mg/kg) significantly increased the number of entries (*p* ≤ 0.05) and the time spent (*p* ≤ 0.001) in the open arms from 1.4 ± 0.55 and 16.75 ± 4.27 s in the negative control (CRS) to 6.20 ± 0.84 and 188.2 ± 13.59 s respectively (**Table 1**). As awaited, the diazepam caused a more significant increase in number of entries (*p* ≤ 0.001) and time spent (*p* ≤ 0.001) in the open arms. The co-administration of diazepam (3 mg/kg) and AEGD (15 mg/kg) also caused a significant increase in number of entries (*p* ≤ 0.05) in the open arms (**Table 1**).

**Table 1:** Effects of the AEGD on the anxiety-related behavior in the elevated plus maze.

Treatment	Open arm entries	Open arm time (s)	Close arm entries	Close arm time (s)	Total number of entries
Vehicle	2.8 0.8	72.6 13.3ˆa	5.0 1.0	223.3 10.7	7.8 1.1
Vehicle + CRS	1.4 0.5	16.7 4.3ˆ*	2.4 0.5	281.7 5.1	3.8 0.8
DZP + CRS	8.2 0.4ˆa*	178.0 9.82ˆb**	5.8 1.5	117.0 9.4ˆb*	14.0 1.2ˆa
GD 7.5 mg/kg + CRS	6.2 0.8	188.2 13.6ˆb**	4.4 0.5	111.8 13.6ˆb*	10.6 0.9ˆa
GD 15 mg/kg + CRS	7.4 0.5	98.4 6.5ˆa	4.2 0.4	196.0 4.0ˆa	11.6 0.5ˆa
GD15 mg/kg + DZP + CRS	10.6 0.5ˆa*	83.8 4.8ˆa	4.0 0.7	212.0 5.2	14.6 0.9ˆa

*Results are expressed as mean ± SEM for the number of entries in the open arm and in the close arm; the time spent in the open arms and in the close arm and the total number of entries. *N* = 5 per group. Data were analyzed using one-way ANOVA, followed by Newman–Keuls *post hoc* test and corrected with Bonferroni multiple comparisons test. ^∗^*p* < 0.05, ^∗∗^*p* < 0.01 *vs* vehicle (distilled water); ^a^*p* < 0.05, ^b^*p* < 0.01 *vs* negative control (vehicle + CRS). CRS, chronic restraint stress; vehicle, distilled water (10 ml/kg, *p.o*.); GD, *G. dalenii* (7.5 and 15 mg/kg, *p.o.*); DZP, diazepam (3 mg/kg, *i.p.*).*

**FIGURE 1:**
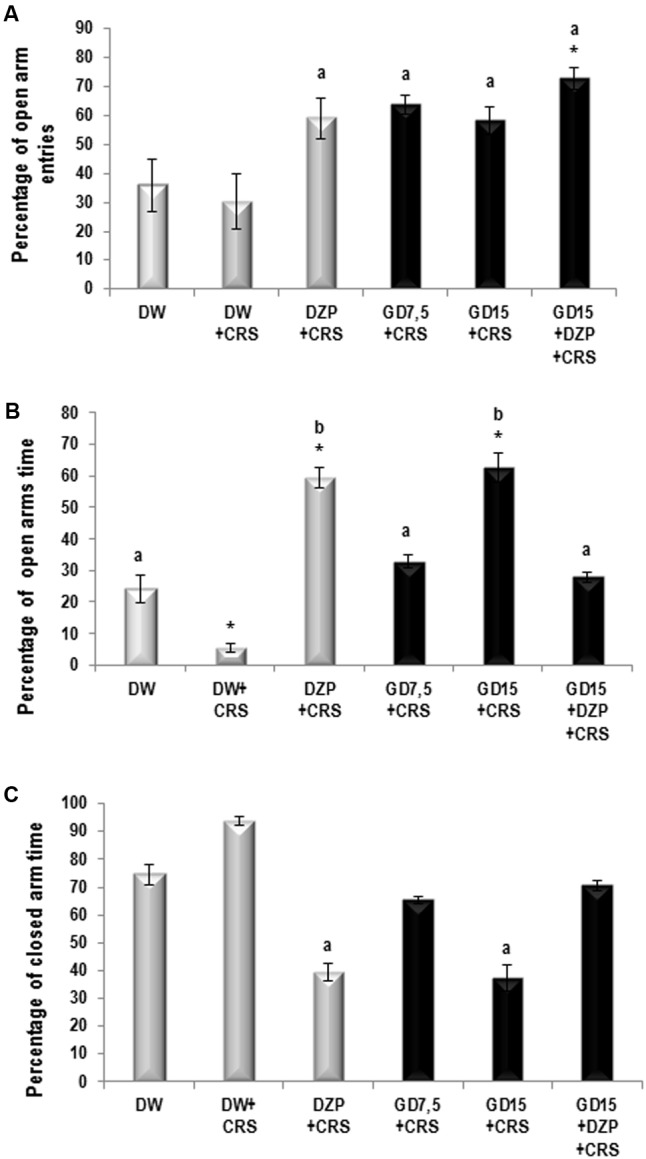
**(A)** Percentage of open arm entries. **(B)** Percentage of open arm time. **(C)** Percentage of closed arm entries. Each bar represents the mean ± SEM for the percentage of open arm entries, percentage of open arm time, and percentage of close arm entries. *N* = 5 per group. Data were analyzed using one-way ANOVA, followed by Newman–Keuls *post hoc* test and corrected with Bonferroni multiple comparisons test. ^∗^*p* < 0.05, *vs* control (distilled water); ^a^*p* < 0.05, ^b^*p* < 0.01, *vs* negative control (DW + CRS). CRS, chronic restraint stress; GD, *G. dalenii* (7.5 and 15 mg/kg, *p.o.*); DZP, diazepam (3 mg/kg, *i.p.*).

The CRS caused a decrease (not significant) of the percentage of the open arm entries. The percentage of entries in the open arms significantly (*p* ≤ 0.05) increased from 30.33 ± 9.31% in the stressed group to 58.39 ± 5.81 and 63.79 ± 3.4% in the groups treated with AEGD at the dose of 15 and 7.5 mg/kg, respectively. As anticipated, diazepam significantly (*p* ≤ 0.001) increased the open arm entries percentage to 59.01 ± 7% (**Figure 1A**). The CRS caused a significant (*p* ≤ 0.005) decrease of the percentage of time spent in the open arm. After the treatments, the percentage of open arm time increased significantly (*p* ≤ 0.01) from 5.58 ± 1.42% in the negative control group to 59.33 ± 3.27 and 62.73 ± 4.53% in the diazepam and the AEGD at the dose of 15 mg/kg treated groups, respectively (**Figure 1B**). On the other hand, the CRS caused an increase (not significant) of the percentage of close arm time. The AEGD at the dose of 15 mg/kg and the diazepam (3 mg/kg) showed a significant (*p* ≤ 0.005) decrease of the percentage of close arm time to 37.27 ± 4.53 and 39.31 ± 3.1%, respectively (**Figure 1C**). This stress-induced behavioral change could be attributed to an imbalance in the neuroendocrine system (Rai et al., 2003; Kenjale et al., 2007).

### Effect of AEGD on Stress-Induced Neurochemical Changes

#### Effects of AEGD on the Plasma Adrenaline and Serotonin Corticosterone and Prolactin Levels

Restraint stress group showed significant (*p* ≤ 0.01) decrease in the level of serum serotonin when compared with vehicle control. Groups treated with AEGD (15 mg/kg) + diazepam (3 mg/kg) along with the restraint stress showed significant (*p* ≤ 0.05) increase in the levels of serotonin when compared with restraint stress group (**Table 2**). The significant decrease (*p* ≤ 0.05) in adrenaline level in negative control group (CRS) compared with vehicle control was significantly (*p* ≤ 0.05) increased in the groups that received AEGD (15 mg/kg), and diazepam + AEGD (15 mg/kg) along with the restraint stress when compared with restraint stress group (**Table 2**).

**Table 2:** Effects of the AEGD on the adrenaline, serotonin, corticosterone, and prolactin levels.

	Biochemical parameters			
Treatment	Serotonin (μg/100 ml)	Adrenaline (μg/100 ml)	Prolactin (ng/ml)	Corticosterone (μg/100 ml)
Vehicle	21.40 2.54ˆa	10.66 1.20ˆa	3.42 0.09ˆa	3.12 1.18ˆa
Vehicle + CRS	11.28 0.86ˆ*	3.52 1.0ˆ*	8.52 0.09ˆ*	8.52 2.21ˆ*
DZP + CRS	16.34 1.21	5.26 1.6	4.36 0.16	3.45 0.68ˆa
GD 7.5 mg/kg + CRS	14.88 0.44	4.65 0.54	6.32 0.16	5.35 0.39
GD 15 mg/kg + CRS	16.33 1.22	6.02 0.33ˆa	5.77 0.12	4.33 0.76
GD15 mg/kg + DZP + CRS	17.81 0.49ˆa	7.21 0.94ˆa	4.23 0.06	3.5 0.33ˆa

*Results are expressed as mean ± SEM for the levels of adrenaline, serotonin, corticosterone, and prolactin. *N* = 5 per group. Data were analyzed using one-way ANOVA, followed by Newman–Keuls *post hoc* test and corrected with Bonferroni multiple comparisons test. ^∗^*p* < 0.05 *vs* vehicle (distilled water); ^a^*p* < 0.05 *vs* negative control (vehicle + CRS). CRS, chronic restraint stress; vehicle, distilled water (10 ml/kg, *p.o*.); GD, *G. dalenii* (7.5 and 15 mg/kg, *p.o.*); DZP, diazepam (3 mg/kg, *i.p.*).*

Restraint stress group showed significant (*p* ≤ 0.05) increase in the level of serum prolactin when compared with vehicle control. Group that received AEGD (15 mg/kg) + diazepam (3 mg/kg) along with the restraint stress showed significant (*p* ≤ 0.05) decrease in the levels of prolactin when compared with restraint stress group (**Table 2**). The significant (*p* ≤ 0.05) increased in the corticosterone level in negative control group (CRS) compared to vehicle control was significantly (*p* ≤ 0.05) reduced in the groups that received AEGD (15 mg/kg) and AEGD (15 mg/kg) + diazepam (3 mg/kg) along with the restraint stress (**Table 2**).

#### Effects of AEGD on the Plasma Progesterone, Estradiol, FSH, and LH Levels

Restraint stress group showed a significant (*p* ≤ 0.05) increase in the level of serum progesterone when compared with vehicle control. Groups that received AEGD (15 mg/kg) body weight, diazepam, and diazepam + AEGD (15 mg/kg) along with the restraint stress showed a decrease (not significant) in the levels of progesterone when compared with restraint stress group (**Table 3**).

**Table 3:** Effects of the AEGD on the estradiol, FSH, LH, and progesterone hormones levels.

Treatment	Progesterone (ng/ml)	Estradiol (pg/ml)	FSH (ng/ml)	LH (ng/ml)
Vehicle	48.65 4.30ˆa	28.10 3.02ˆa	194.79 7.45ˆa	23.01 1.89ˆa
Vehicle + CRS	76.84 7.00ˆ*	14.08 4.07ˆ*	159.87 13.90ˆ*	5.01 0.81ˆ*
DZP + CRS	51.01 4.20	21.75 2.13ˆa	182.62 7.80ˆa	19.19 1.30ˆa
GD 7.5 mg/kg + CRS	62.3 1.73	18.57 0.41	174.54 3.44	16.86 1.13
GD 15 mg/kg + CRS	56.23 1.15	19.42 0.85	182.37 2.15ˆa	19.10 0.14ˆa
GD15 mg/kg + DZP + CRS	52.44 2.57	22.48 1.52ˆa	189.83 1.56ˆa	20.75 0.51ˆa

*Results are expressed as mean ± SEM for the levels of estradiol, FSH, LH, and progesterone. *N* = 5 per group. Data were analyzed using one-way ANOVA, followed by Newman–Keuls *post hoc* test and corrected with Bonferroni multiple comparisons test. ^∗^*p* < 0.05 *vs* vehicle (distilled water); ^a^*p* < 0.05 *vs* negative control (vehicle + CRS). CRS, chronic restraint stress; vehicle, distilled water (10 ml/kg, *p.o.*); GD, *G. dalenii* (7.5 and 15 mg/kg, *p.o.*); DZP, diazepam (3 mg/kg, *i.p.*).*

The restraint stress group exhibited significant drop in the level of serum estradiol when compared with vehicle control. Groups that received diazepam (3 mg/kg) and diazepam + AEGD (15 mg/kg) along with the restraint stress showed significant (*p* ≤ 0.05) increase in the levels of estradiol when compared with restraint stress group (**Table 3**).

The assessment of the FSH levels reveals that restraint stress group showed a significant (*p* ≤ 0.05) decrease in the level of serum FSH when compared with vehicle control. AEGD (15 mg/kg), diazepam (3 mg/kg), and diazepam + AEGD (15 mg/kg) significantly (*p* ≤ 0.05) reversed the reduction of FSH induced by the CRS (**Table 3**).

The restraint stress group showed significant (*p* ≤ 0.05) decrease in the level of serum LH when compared with vehicle control. Groups that received AEGD (15 mg/kg), diazepam, and diazepam + AEGD (15 mg/kg) along with the restraint stress showed significant (*p* ≤ 0.05) increase in the levels of LH when compared with restraint stress group (**Table 3**).

#### Effects of AEGD on the Plasma Cholesterol, Triglycerides, and Glucose Levels

Assessment of the cholesterol levels revealed that CRS significantly (*p* ≤ 0.05) increased the levels of cholesterol compared with the vehicle control rats. Groups that received diazepam (3 mg/kg) and diazepam + AEGD (15 mg/kg) along with the restraint stress showed a significant increase in cholesterol levels when compared with restraint stress group (**Table 4**). Restraint stress group showed significant (*p* ≤ 0.05) decrease in the level of serum triglycerides when compared with vehicle control. Groups that received diazepam (3 mg/kg) and diazepam (3 mg/kg) + AEGD (15 mg/kg) along with the restraint stress showed significant (*p* ≤ 0.05) increase in the concentration of triglycerides when compared with restraint stress group (**Table 4**). Stress rats showed highly significant (*p* ≤ 0.01) decrease in the level of serum glucose when compared with vehicle control. Groups that received AEGD (15 mg/kg), diazepam (3 mg/kg), and diazepam + AEGD (15 mg/kg) along with the restraint stress showed significant (*p* ≤ 0.05) increase in the levels of glucose when compared with restraint stress group (**Table 4**). This stress-induced neurochemical imbalance could lead to reproductive impairments (Anderson et al., 1996).

**Table 4:** Effect of the AEGD on the cholesterol, triglycerides, and glucose levels.

Treatments	Biochemical parameters (mg/dl)		
	Cholesterol	Triglycerides	Glucose
Vehicle	68.85 0.85ˆa	95.56 0.69ˆa	128.35 1.46ˆb
Vehicle + CRS	39.05 1.07ˆ*	71.46 1.24ˆ*	76.25 0.93ˆ**
DZP + CRS	65.92 1.68ˆa	93.57 1.12ˆa	113.79 1.12ˆa
GD 7.5 mg/kg + CRS	54.16 2.79	81.14 0.87	90.26 0.72ˆ*
GD 15 mg/kg + CRS	60.71 0.54	90.66 1.11	100.56 1.87ˆa
GD15 mg/kg + DZP + CRS	66.78 1.12ˆa	93.66 1.18ˆa	117.41 2.41ˆa

*Results are expressed as mean ± SEM for the levels of cholesterol, triglycerides, and glucose. *N* = 5 per group. Data were analyzed using one-way ANOVA, followed by Newman–Keuls *post hoc* test and corrected with Bonferroni multiple comparisons test. ^∗^*p* < 0.05, ^∗∗^*p* < 0.01 *vs* vehicle (distilled water); ^a^*p* < 0.05, ^b^*p* < 0.01 *vs* negative control (vehicle + CRS). CRS, chronic restraint stress; vehicle, distilled water (10 ml/kg, *p.o.*); GD, *G. dalenii* (7.5 and 15 mg/kg, *p.o.*); DZP, diazepam (3 mg/kg, *i.p.*).*

### Effect of AEGD on Stress-Induced Reproductive Changes

#### Effect of AEGD on Stressed Rat’s Estrous Cycle

Stressed animals exhibited changes in the mean duration of estrous cycle phases when compared to vehicle control. It was recorded a significant (*p* ≤ 0.05) increase in proestrus phase length and significant (*p* ≤ 0.05) decreases in estrous and metestrus phases duration. Groups that received AEGD (15 mg/kg), diazepam (3 mg/kg), and diazepam (3 mg/kg) + AEGD (15 mg/kg) along with the restraint stress showed a significant (*p* ≤ 0.05) restoration of proestrus, estrous, and metestrus duration compared to stressed groups (**Table 5**).

**Table 5:** Effects of AEGD on mean numbers of days on different phases of estrous cycle (28 days).

Treatment	Proestrus (days)	Estrous (days)	Metestrus (days)	Diestrus (days)
Vehicle	4.37 0.19ˆa	6.90 0.57	5.99 0.18ˆa	10.78 0.35
Vehicle + CRS	15.58 0.40ˆ*	2.11 0.26ˆ*	1.63 0.20ˆ*	8.05 0.23
DZP + CRS	4.12 0.06ˆa	5.52 0.29	6.47 1.29	11.35 0.04
GD 7.5 mg/kg + CRS	6.03 0.31	4.51 0.35	6.95 0.48ˆa	9.59 0.38
GD 15 mg/kg + CRS	4.99 0.32ˆa	6.91 0.47ˆa	5.14 0.47	10.16 0.45
GD15 mg/kg + DZP + CRS	4.00 1.00ˆa	6.40 0.55ˆa	6.40 0.55ˆa	10.80 0.84

*Results are expressed as mean ± SEM for the mean number of days of proestrus, estrous, metestrus, and diestrus. *N* = 5 per group. Data were analyzed using one-way ANOVA, followed by Newman–Keuls *post hoc* test and corrected with Bonferroni multiple comparisons test. ^∗^*p* < 0.05 *vs* vehicle (distilled water); ^a^*p* < 0.05 *vs* negative control (vehicle + CRS). CRS, chronic restraint stress; vehicle, distilled water (10 ml/kg, *p.o.*); GD, *G. dalenii* (7.5 and 15 mg/kg, *p.o.*); DZP, diazepam (3 mg/kg, *i.p.*).*

#### Effect of the AEGD on the Different Organs Weights

Restraint stress group showed significant (*p* ≤ 0.05) decrease in the adrenal glands weight when compared with vehicle control. Groups that received AEGD (7.5 mg/kg), AEGD (15 mg/kg), diazepam, and diazepam + AEGD (15 mg/kg) along with the CRS showed increase (not significant) in the weight of the adrenal gland when compared with restraint stress group. The CRS also caused a significant decrease in uteri weight compared to vehicle control. Diazepam (3 mg/kg), and diazepam + AEGD (15 mg/kg) significantly (*p* ≤ 0.05) reversed the uteri weight loss compared to stress rats (**Table 6**).

**Table 6:** Effect of the AEGD on the different organs weights (g) in the restraint stressed rats.

Treatment	Adrenal gland	liver	Ovaries	Uterus	Adrenal gland
Vehicle	0.010 ± 0.0006^a^	3.37 ± 0.04	0.03 ± 0.0012	0.19 ± 0.001^a^	0.010 ± 0.0006^a^
Vehicle + CRS	0.019 ± 0.0001^∗^	2.50 ± 0.02	0.02 ± 0.0006	0.11 ± 0.002^∗^	0.019 ± 0.0001^∗^
DZP + CRS	0.010 ± 0.0004	3.54 ± 0.22	0.03 ± 0.0008	0.18 ± 0.003^a^	0.010 ± 0.0004
GD 7.5 mg/kg + CRS	0.010 ± 0.0004	3.31 ± 0.42	0.03 ± 0.0011	0.17 ± 0.001	0.010 ± 0.0004
GD 15 mg/kg + CRS	0.010 ± 0.0008	3.15 ± 0.04	0.14 ± 0.1510^a∗^	0.14 ± 0.069	0.010 ± 0.0008
GD15 mg/kg + DZP + CRS	0.010 ± 0.0006	3.37 ± 0.00	0.03 ± 0.0013	0.21 ± 0.015^a^	0.010 ± 0.0006

*Results are expressed as mean ± SEM for the weight of liver, ovaries, uterus, and adrenal gland. *N* = 5 per group. Data were analyzed using one-way ANOVA, followed by Newman–Keuls *post hoc* test and corrected with Bonferroni multiple comparisons test. ^∗^*p* < 0.05 *vs* vehicle (distilled water); ^a^*p* < 0.05 *vs* negative control (vehicle + CRS). CRS, chronic restraint stress; vehicle, distilled water (10 ml/kg, *p.o.*); GD, *G. dalenii* (7.5 and 15 mg/kg, *p.o.*); DZP, diazepam (3 mg/kg, *i.p.*).*

#### Effect of AEGD on the Ovary Histological Analysis of the Treated Rats

**FIGURE 2:**
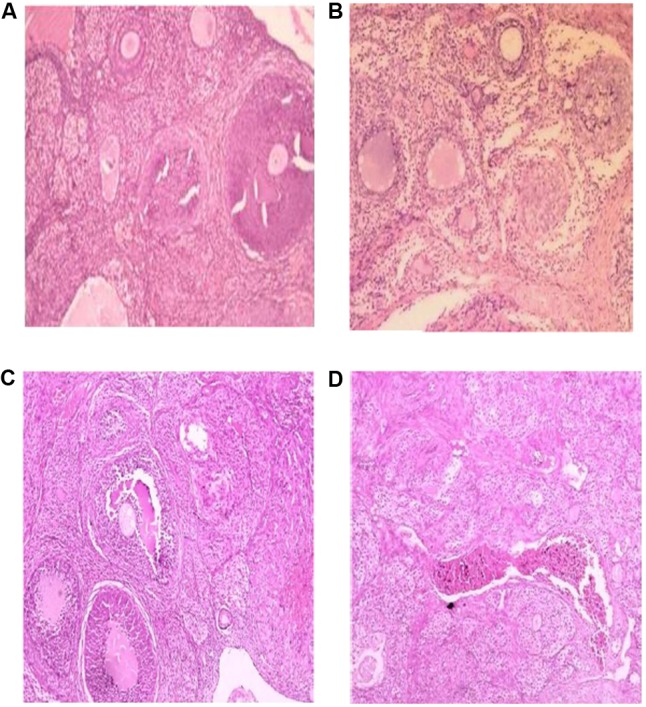
Effect of AEGD on the ovary histological analysis of the treated rats (hematoxylin–eosin, 100×). **(A)** Section of (vehicle control, distilled water) rat ovary showing normal stroma with primary and secondary developing follicles and matured graafian follicle. **(B)** Section of (chronic restraint stress) rat ovary showing hyperchromatic nucleus, multiple follicular cysts and atretic follicles and corpus fibrosum. **(C)** Section of (chronic restraint + *G. dalenii* 7.5 mg/kg) rat ovary showing only few developing follicles. **(D)** Section of (chronic restraint stress + *G. dalenii* 15 mg/kg) rat ovary showing most follicles with normal follicular development and intact corpus luteum.

The histological analysis of the ovaries revealed localized alterations of tissues in stressed rats (**Figure 2A**) with hyperchromatic nucleus, multiple follicular cysts and atretic follicles and corpus fibrosum compared to the vehicle control group (**Figure 2B**) that had a normal stroma with primary and secondary developing follicles and matured graafian follicle. In the stressed rats and treated with the AEGD at the dose 7.5 mg/kg (**Figure 2C**) and AEGD at the dose of 15 mg/kg (**Figure 2D**), the changes observed in the stress rats were reversed with a normal stroma, developing follicles, and matured graafian follicle.

## Discussion

The results of this study indicate that in the open arms, the number of entries, the time spent and the respective percentages significantly increased in the chronic restraint stressed non-epileptic adult female rats, in the presence of the AEGD at doses of 7.5 and 15 mg/kg and were comparable to the effects of diazepam a recognized anxiolytic dose (3 mg/kg). On the contrary the AEGD significantly decreased the percentage of closed arms entries and time spent. Any increased activity in open arms indicates a decreased anxiety level (Lee and Rodgers, 1991; Rodgers and Dalvi, 1997; Holmes et al., 2000; Majchrzac, 2003). Also, a decrease of these behavioral parameters in the closed arms indicates a reduction of stress level (Lister, 1990; Ngo Bum et al., 2009). These results show the anxiolytic-like activity of the AEGD (Gomes et al., 2010; Souto-Maior et al., 2011). Diazepam is referred to as an anxiolytic in humans and causes decrease in anxiogenic-like. Several studies have reported that diazepam at anxiolytic dose facilitates exploratory behavior which is expressed as increased locomotion in the elevated plus maze (Bhattacharya and Mitra, 1991; Ramanathan et al., 1998). Our findings showed that the animals treated with the AEGD at the doses of 7.5 and 15 mg/kg caused increase in the opened arm entries without increasing the total number of entries thereby leading to not changes in locomotion of rats. In order to further corroborate the anxiolytic activity observed in the EPM test, we also assessed the stress markers levels. Our results showed that stressed rats exhibited anxiogenic behavior associated to reduction of plasma adrenaline and serotonin concentrations and the increased plasma corticosterone, progesterone, and prolactin levels. This reveals that the rats underwent stress and the alteration observed is similar to clinically related pathophysiology of anxiety (Saavedra et al., 1979; Saavedra and Torda, 1980; Heim and Nemeroff, 1999; Millan, 2003). Administration of the AEGD during stress period restored the exploratory behavior of rats. The increased exploratory behavior of rats was correlated with restoration of plasma adrenaline levels (Srinivasan et al., 2003). The results showed that stressed rats treated with the AEGD had corticosterone level significantly reduced almost to normal values (Kyrou and Tsigos, 2009). The HPA axis is made up of an assembly of stress responses mediated by the brain, pituitary, and adrenal gland. The endocrine activity of the hypothalamus causes the production of the CRF, a compound that stimulates the production of adrenocorticotropic hormone (ACTH). ACTH is liberated into the circulatory system, and causes the adrenal cortex to secrete corticosteroid hormones, particularly cortisol. Cortisol increases the availability of refueling the body with substances necessary for the body’s response to stress (Dornhorst et al., 1981). The results showed a significant drop in glucose and triglycerides levels in stressed rats when compared to vehicle control group. These substances levels were reversed and were returned to more normal value in AEGD treated stressed rats, this suggests that the AEGD showed anxiolytic properties. These findings are similar to the results obtained by Fotsing et al. (2016) in the analogous studies with *N. canescens.* The AEGD is rich in polyphenols, flavonoids, tannins, tripertenes, or other secondary metabolites that may support the anxiolytic activity of the plant (Harsha and Anilakumar, 2013a,b; Ngoupaye et al., 2013a). Anti-anxiety secondary metabolites can interfere with the serotonin and GABA systems; this may explain the similar effects of the AEGD and diazepam, a GABA benzodiazepine agonist, in relieving anxiety (Pravinkumar et al., 2007; Priprem et al., 2008).

To further establish the anxiolytic properties of the AEGD, we studied its effects on the reproductive parameters. Because stress can alter neurotransmitters and hormones involved in the regulation of reproductive physiology, it has been reported that stress affects reproductive function in female (Anderson et al., 1996). Chronic restrained rats showed a significant rise in the mean number of days in proestrus phase and decrease in estrous and metestrus phases (Brotman et al., 2007). This demonstrates the disruption of follicular development at the initial stages causing the non-maturation of follicles (Sivaprasad et al., 2015). The AEGD treated groups showed significant decrease duration of proestrus phase indicating the development of follicles. The treatment also causes a significant increase in the mean days of estrous, metestrus, and diestrus phases. These findings reveal the antagonizing effect of the AEGD against stress-induced estrous cycle changes. It also indicates the maturation of follicles, the formation of Graafian follicles and corpus luteum due to the increased secretion of either gonadotrophic, or steroidal hormones or both (Bhutani et al., 2004). The ovaries are made up of three endocrine tissues, the stroma, the follicle and the corpus luteum. Therefore, the net weight of the ovaries is the sum of the weights of these tissues. Our study showed that there was a decrease in the ovarian weight in stressed rats. This undoubtedly indicated that there was no follicular development and consequently decreased activities of the stroma, the follicles, and the corpus luteum caused by non-availability of either gonadotrophic hormones or the steroidal hormones or both (Shivalingappa et al., 2002). Concerning the effect of CRS on the pituitary–ovarian axis of the adult female albino rats, the present investigation showed a significant reduction in serum FSH, LH, and estradiol concentrations. Moreover, ovarian histological changes were detected in the stressed rats as evidenced with the hyperchromatic nucleus, multiple follicular cysts, atretic follicles, and corpus fibrosum when compared with the distilled water treated rats. The treatment with the AEGD displayed protective effects on the ovaries that showed a normal stroma, developing follicles and matured graafian follicle. The chronic immobilization stress also caused a significant decrease in uterine weight and this was caused by the non-availability of hormones required for the development of the uterus (Fotsing et al., 2016). AEGD treated groups showed prevention in the loss of weight of uterus which may be due to uterotrophic effect of the plant. The significant increased weight of adrenal glands in stress rats is related to the active involvement of the HPA and sympathetic stimulation, which is fast to respond to stress.

## Conclusion

In this work, the effects of AEGD on the chronic immobilization stress-induced behavioral, neurochemical, and reproductive changes in female albino rats were assessed. The findings revealed that the AEGD significantly increased the number of entries and the time spent in the open arm of the EPM. The chronic immobilization stress-induced increased corticosterone, progesterone, and prolactin concentrations were antagonized by *G. dalenii*. Moreover, the decreases in reproductive hormones as well as the changes in estrous cycle duration caused by the chronic immobilization stress were normalized in the *G. dalenii* treated rats. AEGD displayed adaptogenic potential against chronic restraint model on experimental animals. Further studies may be carried out to identify and characterize the active principles and their mechanism of action.

## Ethics Statement

This study was carried out in accordance with the recommendations of the Cameroon National Ethical Committee (Ref No. FW-IRB00001954). The protocol was approved by the Comité d’Ethique Institutionnel de la Recherche pour la Santé Humaine with an authorization number CEI-UDo/909/01/2017/T.

## Author Contributions

DF, ENB, SN, and YK made substantial contributions to the conception or design of the work. DF, ENB, SN, and YK contributed to the acquisition and analysis of the data. DF, ENB, SN, GN, and AO interpreted data for the work. DF, ENB, GN, and AO drafted the work. DF, ENB, YK, and GN critically revised for important intellectual content. DF, ENB, YK, and GN approved the final version to be published. DF, ENB, SN, YK, and AO agreed to be accountable for all aspects of the work in ensuring that questions related to the accuracy. DF, ENB, YK, and GN agreed to be accountable for integrity of any part of the work are appropriately investigated and resolved.

## Conflict of Interest Statement

The authors declare that the research was conducted in the absence of any commercial or financial relationships that could be construed as a potential conflict of interest.

## References

[B1] Adejuwon A. S. Femi-Akinlosotu O. M. Omirinde J. O. (2013). Effect of graded doses of aqueous extract of *Gladiolus dalenii* on the semen parameters of wistar rats. *Int. J. Indig. Med. Plants* 46 1191–1195.

[B2] Anderson S. A. Saviolakis G. A. Bauman R. A. Chu K. Y. Ghosh S. Kant G. J. (1996). Effects of chronic stress on food acquisition, plasma hormones, and the estrous cycle of female rats. *Physiol. Behav.* 60 325–329. 10.1016/0031-9384(96)00023-6 8804685

[B3] Baron S. Brush F. R. (1979). Effects of acute and chronic restraint and estrus cycle on pituitary-adrenal function in the rat. *Horm. Behav.* 12 218–224. 10.1016/0018-506X(79)90004-7 575714

[B4] Bhattacharya S. K. Mitra S. K. (1991). Anxiolytic activity of *Panax ginseng* roots: an experimental study. *J. Ethnopharmacol.* 34 87–92. 10.1016/0378-8741(91)90193-H 1684404

[B5] Bhutani K. K. Jadhav A. N. Kalia V. (2004). Effects of *Symplocos racemosa* Roxb on gonadotropin release in immature female rats and ovarian histology. *J. Ethnoparmacol.* 94 197–200. 10.1016/j.jep.2004.04.022 15261983

[B6] Bowman R. E. Zrull M. C. Luine V. L. (2001). Chronic restraint stress enhances radial arm maze performance in female rats. *Brain Res.* 904 279–289. 10.1016/S0006-8993(01)02474-X 11406126

[B7] Brotman D. J. Golden S. H. Wittstein I. S. (2007). The cardiovascular toll of stress. *Lancet* 370 1089–1100. 10.1016/S0140-6736(07)61305-1 17822755

[B8] Campo-Soria C. Yongchang C. David S. W. (2006). Mechanism of action of benzodiazepines on GABAA receptors. *Br. J. Pharm.* 148 984–990. 10.1038/sj.bjp.0706796 PMC1751932 16783415

[B9] Chatterjee A. Rajikin M. H. Chatterjee R. Sumitabha G. (2006). Mini review: stress and how it affects reproduction. *Biomed. Res.* 17 1–6.

[B10] Czobor P. Skolnick P. Beer B. Lippa A. (2010). A multicenter, placebo-controlled, double-blind, randomized study of efficacy and safety of ocinaplon (DOV 273, 547) in generalized anxiety disorder. *CNS Neurosci. Ther.* 16 63–75. 10.1111/j.1755-5949.2009.00109.x 20041911 PMC6493815

[B11] Dornhorst A. Carlson D. E. Seif S. M. Robinson A. D. Zimmerman D. E. Gann D. S. (1981). Control of release of adrenocorticotropin and vasopressin by the supraoptic and paraventricular nuclei. *Endocrinology* 108 1420–1424. 10.1210/endo-108-4-1420 6258907

[B12] Fenster L. Waller K. Chen J. Hubbard A. E. Windham G. C. Elkin E. (1999). Psychological stress in the workplace and menstrual function. *Am. J. Epidemol.* 149 127–134. 10.1093/oxfordjournals.aje.a009777 9921957

[B13] Fotsing D. Njapdounke K. J. Kenneth Y. A. Ngo Bum E. (2016). Effect of *Nelsonia canescens* (Acanthaceae) on the stress induced behavioral and reproductive changes in female rats. *World J. Pharm. Pharm. Sci.* 12 31–49. 10.20959/wjpps201612-8154

[B14] Fredericksen S. O. Ekman R. Gottfries C. G. Widerlöv E. Jonsson S. (1991). Reduced concentration of galanin, arginine vasopressin, neuropeptide Y and peptide YY in the temporal cortex but not the hypothalamus of brains from schizophrenics. *Acta Psychiatr. Scand.* 83 273–277. 10.1111/j.1600-0447.1991.tb05539.x 1709331

[B15] Genazzani A. D. Ricchieri F. Lanzoni C. Strucchi C. Jasonni V. M. (2006). Diagnostic and therapeutic approach to hypothalamic amenorrhea. *Ann. N. Y. Acad. Sci.* 1092 103–113. 10.1196/annals.1365.009 17308137

[B16] Gomes P. B. Feitosa M. L. Silva M. I. Noronha E. C. Moura B. A. Venancio E. T. (2010). Anxilytic-like effect of the monopertene 1,4-cineole in mice. *Pharmacol. Biochem. Behav.* 96 287–293. 10.1016/j.pbb.2010.05.019 20670917

[B17] Harsha S. N. Anilakumar K. R. (2013a). Anxiolytic property of *Lactuca sativa*, effects on anxiety behaviour induced by novel food and height. *Asian Pac. J. Trop. Med.* 6 532–536. 10.1016/S1995-7645(13)60091-7 23768824

[B18] Harsha S. N. Anilakumar K. R. (2013b). Anxiolytic property of hydro-alcohol extract of *Lactuca sativa* and its effects on behavioral activities of mice. *J. Biomed. Res.* 27 37–42. 10.7555/JBR.27.20120059 23554792 PMC3596753

[B19] Heim C. Nemeroff C. B. (1999). The impact of early adverse experiences on brain systems involved in the pathophysiology of anxiety and affective disorders. *Biol. Psychiatry* 46 1509–1522. 10.1016/S0006-3223(99)00224-3 10599479

[B20] Holmes A. Parmigiani S. Ferrari P. F. Palanza P. Rodgers R. J. (2000). Behavioural profile of wild mice in the elevated plus-maze test for anxiety. *Physiol. Behav.* 71 509–516. 10.1016/S0031-9384(00)00373-5 11239669

[B21] Horrocks P. M. Jones A. F. Ratcliffe W. A. Holder G. White A. Holder R. (1990). Pattern of ACTH and cortisol pulsatility over twenty-four hours in normal males and females. *Clin. Endocrinol.* 32 127–134. 10.1111/j.1365-2265.1990.tb03758.x 2158868

[B22] Imaki T. Naruse M. Harada S. Chikada N. Imaki J. Onodera H. (1996). Corticotropin releasing factor up-regulates its own receptor m-RNA in the paraventricular nucleus of the hypothalamus. *Brain Res. Mol. Brain Res.* 38 166–170. 10.1016/0169-328X(96)00011-3 8737681

[B23] Kaplan J. R. Manuck S. B. (2004). Ovarian function, stress and disease: a primate continuum. *ILAR J.* 45 89–97. 10.1093/ilar.45.2.89 15111730

[B24] Kenjale R. Shah R. Sataye S. (2007). Anti-stress and anti-oxidant effects of roots of *Chlorophytum borivilianum*. *Indian J. Exp. Biol.* 12 974–979. 18072542

[B25] Kyrou I. Tsigos C. (2009). Stress hormones: physiological stress and regulation of metabolism. *Curr. Opin. Pharmacol.* 9 787–193. 10.1016/j.coph.2009.08.007 19758844

[B26] Lader M. H. Morton S. (1991). The benzodiazepine problems. *Br. J. Addict.* 86 823–828. 10.1111/j.1360-0443.1991.tb01831.x 1680514

[B27] Lee C. Rodgers R. J. (1991). Effects of buspirone on antinociceptive and behavioural responses to the elevated plus- maze. *Behav. Pharmacol.* 2 491–496. 10.1097/00008877-199112000-00006 11224091

[B28] Lister R. G. (1990). Ethologically based animal models of anxiety disorders. *Pharmacol. Theratol.* 46 321–340. 10.1016/0163-7258(90)90021-S 2188266

[B29] Lund T. D. Munson D. J. Haldy M. E. Handa R. J. (2004). Androgen inhibits, while estrogen enhances, restraint-induced activation of neuropeptide neurones in the paraventricular nucleus of the hypothalamus. *J. Neuroendocrinol.* 16 272–278. 10.1111/j.0953-8194.2004.01167.x 15049858

[B30] Majchrzac M. (2003). Plus maze ou labyrinthe en croix surélevé: livret des techniques. IFR des neurosciences de strasbourg, neurosciences comportementales et cognitives Edité par Tournier B, et Revel F, CNRS/ULP-UMPR7521. *Neurosci. Com. Cogn.* 246 52–89.

[B31] Millan M. J. (2003). The neurobiology and control of anxious states. *Prog. Neurobiol.* 70 83–244. 10.1016/S0301-0082(03)00087-X 12927745

[B32] Nepomnaschy P. A. Welch K. McConnell D. S. Strassman B. I. England B. G. (2004). Stress and female reproductive function: a study of daily variations in cortisol, gonadotrophins, and gonadal steroids in a rural Mayan population. *Am. J. Hum. Biol.* 16 523–532. 10.1002/ajhb.20057 15368600

[B33] Ngo Bum E. Soudi S. Ayissi E. R. Dong C. Lakoulo N. H. Maidawa F. (2011). Anxiolytic activity evaluation of four medicinal plants from Cameroon. *Afr. J. Tradit. Complement. Altern. Med.* 8(5 Suppl.) 130–139. 10.4314/ajtcam.v8i5S.19 22754066 PMC3252713

[B34] Ngo Bum E. Taiwe G. S. Moto F. C. O. Ngoupaye G. T. Nkantchoua G. C. N. Pelanken M. M. (2009). Anticonvulsant, anxiolytic and sedative properties of the roots of *Nauclea latifolia* Smith in mice. *Epilepsy Behav.* 15 434–440. 10.1016/j.yebeh.2009.05.014 19560975

[B35] Ngoupaye G. T. Bum E. N. Daniels W. M. (2013a). Antidepressant like effects of the aqueous macerate of the bulb of *Gladiolus dalenii* Van Geel (Iridacea) in rat model of epilepsy-associated depression. *BMC Complement. Altern. Med.* 13:272 10.1186/1472-6882-13-272 PMC3854025 24138845

[B36] Ngoupaye G. T. Bum E. N. Ngah E. Talla E. Taiwe G. S. Rakotonirina A. (2013b). The anticonvulsivant and sedative effects of *Gladiolus dalenii* extracts in rats. *Epilepsy Behav.* 28 450–456. 10.1016/j.yebeh.2013.06.014 23891766

[B37] Odhiambo J. Sibo G. Lukhoba C. Dossaji S. (2010). Antifungal activity of crude extracts of *Gladiolus dalenii* Van Geel (Iridaceae). *Afr. J. Tradit. Complement. Altern. Med.* 7 53–58. 10.4314/ajtcam.v7i1.57254 PMC3005389 21304613

[B38] Pellow S. Chopin P. File S. E. Briley M. (1985). Validation of open: closed arm entries in an elevated plus-maze as a measure of anxiety in the rat. *J. Neurosci. Methods* 14 149–167. 10.1016/0165-0270(85)90031-7 2864480

[B39] Pravinkumar S. J. Edwards G. Lindsay D. Redmond S. Stirling J. House R. (2007). Anxiolytic effects of *Lavandula angustifolia* odour on the *Mongolian gerbil* elevated plus maze. *J. Ethnoparmacol.* 111 517–525. 10.1016/j.jep.2006.12.021 17289317

[B40] Priprem A. Watanatorn J. Sutthiparinnyanont S. Phachonpai W. Muchimappura S. (2008). Anxiety and cognitive effects of quercetin liposomes in rats. *Nanomedecine* 4 70–78. 10.1016/j.nano.2007.12.001 18249157

[B41] Rai D. Bhatia G. Palit G. Pal R. Singh H. (2003). Adaptogenic effect of *Bacopa monnieri* (Brahmi). *Pharmacol. Biochem. Behav.* 75 823–830. 10.1016/S0091-3057(03)00156-4 12957224

[B42] Ramanathan M. Jaiswal A. K. Bhattacharya S. K. (1998). Differential effects of diazepam on anxiety in streptozotocin induced diabetic and non-diabetic rats. *Psychopharmacology* 135 361–367. 10.1007/s002130050523 9539260

[B43] Rodgers R. J. Dalvi A. (1997). Anxiety defense and the elevated plus maze. *Neurosci. Biobehav. Rev.* 21 801–810. 10.1016/S0149-7634(96)00058-9 9415905

[B44] Saavedra J. M. Kvetnansky R. Kopin I. J. (1979). Adrenaline, noradrenaline and dopamine levels in specific brain stem areas of acutely immobilized rats. *Brain Res.* 160 271–280. 10.1016/0006-8993(79)90424-4 761066

[B45] Saavedra J. M. Torda T. (1980). Increased brain stem and decreased hypothalamic adrenaline-forming enzyme after acute and repeated immobilization stress in the rat. *Neuroendocrinology* 31 142–146. 10.1159/000123065 7393409

[B46] Saraswathi C. D. Sreemantula S. Prakash W. S. (2010). Effect of chronic cold restraint and immobilization stress on estrous cycle in rats. *Pharmacol. Online* 2 151–160.

[B47] Shivalingappa H. Satyanaranyan N. D. Purohit M. G. Sahranabasappa A. Patil S. B. (2002). Effect of ethanol extract of *Rivea hypocrateriformis* on the estrous cycle of the rat. *J. Ethnoparmacol.* 82 11–17. 10.1016/S0378-8741(02)00073-9 12169399

[B48] Sivaprasad G. Upendranadh A. Thirupathi R. K. Ramoji A. (2015). Adaptogenic activity of the ethanolic root extract of *Euphorbia thymifolia* L. in female rats. *Int. Res. J. Pharm.* 6 206–209. 10.7897/2230-8407.06345

[B49] Souto-Maior F. N. De Carvalho F. L. De Morais L. C. Netto S. M. De Sousa D. P. De Almeida R. N. (2011). Anxiolytic-like effects of inhaled linalool oxide in experimental mouse anxiety models. *Pharmacol. Biochem. Behav.* 100 259–263. 10.1016/j.pbb.2011.08.029 21925533

[B50] Srinivasan J. Suresh B. Ramanathan M. (2003). Differential anxiolytic effect of enalapril, and losartan in normotensive, and renal hypertensive rat. *Physiol. Behav.* 78 585–591. 10.1016/S0031-9384(03)00036-2 12782212

[B51] Woods N. F. Lentz M. J. Mitchell E. S. Heitkemper M. Shaver J. Henker R. (1998). Perceived stress, physiologic stress arousal, and premenstrual symptoms: group differences and intra-individual patterns. *Res. Nurs. Health* 21 511–523. 10.1002/(SICI)1098-240X(199812)21:6<511::AID-NUR5>3.0.CO;2-W 9839796

